# Body mass index percentiles versus body composition assessments: Challenges for disease risk classifications in children

**DOI:** 10.3389/fped.2023.1112920

**Published:** 2023-03-03

**Authors:** Jody L. Clasey, Elizabeth A. Easley, Margaret O. Murphy, Stefan G. Kiessling, Arnold Stromberg, Aric Schadler, Hong Huang, John A. Bauer

**Affiliations:** ^1^ Department of Kinesiology and Health Promotion, University of Kentucky, Lexington, KY, United States; ^2^ Department of Pediatrics, University of Kentucky, Lexington, KY, United States; ^3^ Department of Math, Science, Nursing, Public Health, University of South Carolina Lancaster, Lancaster, SC, United States; ^4^ Department of Statistics, University of Kentucky, Lexington, KY, United States

**Keywords:** body mass index, body composition, fat mass index, fat-free mass index, percentage fat

## Abstract

**Background:**

Identifying at-risk children with optimal specificity and sensitivity to allow for the appropriate intervention strategies to be implemented is crucial to improving the health and well-being of children. We determined relationships of body mass indexes for age and sex percentile (BMI%) classifications to actual body composition using validated and convenient methodologies and compared fat and non-fat mass estimates to normative cut-off reference values to determine guideline reliability. We hypothesized that we would achieve an improved ability to identify at-risk children using simple, non-invasive body composition and index measures.

**Methods:**

Cross-sectional study of a volunteer convenience sample of 1,064 (537 boys) young children comparing Body Fat Percentage (BF%), Fat Mass Index (FMI), Fat-Free Mass Index (FFMI), determined *via* rapid bioimpedance methods vs. BMI% in children. Comparisons determined among weight classifications and boys vs. girls.

**Results:**

Amongst all subjects BMI% was generally correlated to body composition measures and indexes but nearly one quarter of children in the low-risk classifications (healthy weight or overweight BMI%) had higher BF% and/or lower FFMI than recommended standards. Substantial evidence of higher than expected fatness and or sarcopenia was found relative to risk status. Inaccuracies were more common in girls than boys and girls were found to have consistently higher BF% at any BMI%.

**Conclusions:**

The population studied raises concerns regarding actual risks for children of healthy or overweight categorized BMI% since many had higher than expected BF% and potential sarcopenia. When body composition and FMI and FFMI are used in conjunction with BMI% improved sensitivity, and accuracy of identifying children who may benefit from appropriate interventions results. These additional measures could help guide clinical decision making in settings of disease-risks stratifications and interventions.

## Introduction

In recent decades the worldwide rate of obesity in children has risen strikingly, especially in settings of lower socioeconomic status (1–5). In 2018 nearly one in five children or adolescents in the US were considered obese, defined as a body mass index (BMI), at or above the 95th percentile of the sex-specific BMI-for-age growth charts (6). Obesity can impact nearly all aspects of a child's life, including risks of several major morbidities (metabolic syndrome, diabetes, cardiovascular diseases, cancer), their psychological health, and their overall physical health (7–13). Childhood obesity is also the major predictor of adult obesity, leading to a continued and often lifetime burden of increased medical and social costs (14, 15). This problem is a major public health concern, and a major driver of medical and social costs, with an estimated financial burden to the US of ∼$14 billion annually.

Overweight and obesity are terms used to describe excess of adiposity, or fatness, above the ideal for good health. Defining obesity severity during childhood requires suitable measures of body fat and appropriate cutoff ranges, especially for the goal of stratifying disease risks, managing clinical care or developing treatment or prevention strategies. This is a common challenge in most pediatric patient care settings, and although according to the Centers for Disease Control and Prevention (CDC) “BMI can be considered a practical alternative to direct measures of body fat”, it has not been highly successful as a tool in children (14, 16). The use of BMI for age and sex percentile (BMI%) alone, or BMI z-score corrected for age and sex, was found to have only modest value in stratifying patients; this is especially true in cases of extreme obesity (15, 17–20). In 2014 Skinner et al. proposed extension obesity classifications to include three gradations, and others have developed similar strategies for improved assessments of individuals with extreme obesity and stratify morbidities risks (5). Although at this time there is no singular defined protocol or universal guideline for assessing obesity status in children there is a clear need to enhance the clarity of which children are of substantial risk of disease (21–23). Equally important is the need for reliable and specific measures to aid in determining treatment effectiveness. While BMI% has often been shown to have generalized predictive value in identifying obese children when compared to BIA, total body dual energy absorptiometry scans and other body composition methodologies, the lack of ability to distinguish between over-fatness or low fat-free mass (particularly in children with a health BMI%) remains problematic (24–27). At this time the pediatric obesity problem is in need of better ways to define which children are of highest concern, and the primary reliance on BMI% may be suboptimal (21).

The goal of this study was to investigate relationships of obesity classification based on current guidelines relative to additional measures of body composition and indexes in a large cohort of school age children for risk stratification. This goal was enabled by a large set of data our institution has accrued *via* several outpatient and/or school-based child body composition assessments, which utilized a reliable and validated set of standardized testing procedures including bioimpedance analysis (BIA) methodologies. The central questions were: how do the current CDC and AAP guidelines for weight classifications compare to actual body composition measurements in a large cohort of school-aged children? And, are there any patterns of deviation amongst boys vs. girls of this age group?

## Materials and methods

### Subjects

Pediatric participants previously enrolled in several clinical research studies conducted in conjunction with the University of Kentucky Pediatric Exercise Physiology Laboratory (PEP Lab) were included in this study. Enrollment criteria included: age 5–11 years, no active health issues, and no known congenital or genetic abnormalities. Data were collected in a variety of settings, including a dedicated clinical research lab for pediatric exercise studies (PEP Lab), and several public elementary schools throughout the state of Kentucky. All instrumentation for body composition measurements and personnel involved in data collection were identical for each subject.

Subject recruitment involved advertisement of the study protocol and/or visitation to a regional public school for description of the research projects, typically days prior to an actual study day to obtain consent/assent. All participants' parents provided written consent and all participants provided verbal assent prior to participation in accordance with the policies, procedures, and approval of the University of Kentucky's Office of Research Integrity Institutional Review Board. No subjects were recruited based on their specific body composition and we consider the enrollment processes employed to be volunteer convenience sampling of our regional population of school age children within the catchment area for our Children's Hospital. We limited our study cohort to 1,064 White Non-Hispanic (WNH) children under the assumption that BMI% may be best suited in this group due to under sampling by the NHANES of other ethnic groups prior to 2011 (28), the greater frequency of participation by WNH children in our clinical research studies, the availability of a pediatric (5–11 years) specific BIA equation that was validated and cross-validated in WNH children only, and the availability of ethnic specific means ± SD for the Fat Mass (FMI) and Fat-free Mass (FFMI) indexes.

In accordance with AAP guidelines, each child was categorized by BMI% using standardized nomogram tools accounting for age and sex, as healthy weight (HW; BMI% >5th, <85th), overweight (OW; BMI% ≥85th <95th), Class I obesity (OB I; BMI% ≥95th <120th), Class II obesity (OB II; BMI% ≥120th <140th), or Class III obesity (OB III; BMI% ≥140th percentile (5, 23, 29).

### Age, anthropometric and body composition measurements

Age was calculated from the date of birth to the testing date. Body mass and standing height were determined using a calibrated digital scale (Escali XL200 Digital Scale; Minneapolis, MN) and a wall-fixed meter stick (Starrett MS-2; Melville, NY) while wearing light-weight clothing and no shoes; these were subsequently used to determine the BMI%. The total body Fat Mass (FM), fat-free mass (FFM), and body fat percentage (BF%) were determined using a tetrapolar bioelectrical impedance analyzer (BIA; Bodystat 4,000 Quadscan; Bodystat, Isle of Man, British Isles). These measures were completed twice (mean of the resulting impedance measures used for subsequent analyses) using previously reported standard procedures, the impedance frequency of 50 kHz, and a validated age and ethnic group specific BIA equation established at our institution (30). In all cases the total time for collection of body composition data *via* these established processes was less than 10 min.

### Reference values for body composition normative comparisons

Body composition variables for each subject were compared to published norms and threshold values currently accepted as appropriate for age and sex (31). We are using these values to represent the ideal or appropriate healthy cutoff values for comparative purposes. The FMI (kg/m^2^) and the FFMI (kg/m^2^) were determined for each child participant by dividing the fat and fat-free body composition mass measures by height in meters squared, and compared to reference measures reported in 2005 by Freedman and colleagues (32). Specifically, these cutoff values were (lower to upper limits): BF%: boys 10%–24%, girls 17%–32%; FMI: boys: 0.5–6.5, girls 1.8–7.8; FFMI: boys 13.2–15.2; girls 12.7–14.7.

### Statistical analyses

Data were analyzed using JMP version 16.0 (SAS Incorporated, Cary, NC) and are presented as means ± standard error. Comparisons of categorical variables were made using Chi-Squared tests. Continuous responses over groups were compared by one-way ANOVA, with normality assumptions justified by the Central Limit Theorem. Regression analyses for variables that were not linearly related employed a quadratic equation including a sex effect. In all cases significance was ascribed at *p* < 0.05.

## Results

Physical characteristics and weight categories of the 1,064 study subjects are shown in Table 1. Average age, weight, height and BMI were not different between the boys (*n* = 537) and girls (*n* = 528) studied. 46% of the boys and 40% of the girls had BMI% classifying them as overweight or obese. Furthermore, 30% of the boys and 24% of the girls had BMI% classifying them as obese (Class I, II and III obesity combined). The distribution of the weight categories for boys vs. girls was not different between groups. More detailed physical characteristics are provided in Supplementary Table S1. The frequency distribution for the BF% and FMI compared to BMI-based classifications (above or below the 95th percentile for age and sex) are shown in Table 2 with resulting sensitivity and specificity of 0.948 and 0.780 for BF%, and 0.902 and 0.952 for the FMI.

**Table 1:** Demographic and weight classification distribution by sex.

	Boys Mean ± SE (range)	Girls Mean ± SE (range)
**Age (years)**	9.6 ± 0.1 (5.0–11.9)	9.7 ± 0.1 (5.0–11.9)
**Body Weight (kg)**	39.4 ± 0.6 (16.5–101.8)	39.1 ± 0.6 (14.4–105.5)
**Standing Height (cm)**	140.0 ± 0.5 (103.8–172.3)	139.9 ± 0.5 (99.4–168.5)
**Body Mass Index (BMI; kg/m^2^)**	19.5 ± 0.2 (13.1–41.4)	19.5 ± 0.2 (12.8–39.7)
**Weight Classifications, *n* (%)**		
Healthy	288 (53%)	314 (59%)
Overweight	90 (17%)	85 (16%)
Obese-I	108 (20%)	89 (17%)
Obese-II	35 (6.5%)	26 (4.9%)
Obese-III	16 (2.9%)	14 (2.7%)
Total	537 (100%)	528 (100%)

**Table 2:** Frequency distribution for the body fat % and the fat-mass index compared to BMI for age and sex percentiles.

	BMI ≥ 95th percentile	BMI < 95th percentile
BF% Reference > excess adiposity reference	272	171
BF% Reference < excess adiposity reference	15	606
FMI > excess adiposity reference	259	37
FMI < excess adiposity reference	28	740

**Figure 1:**
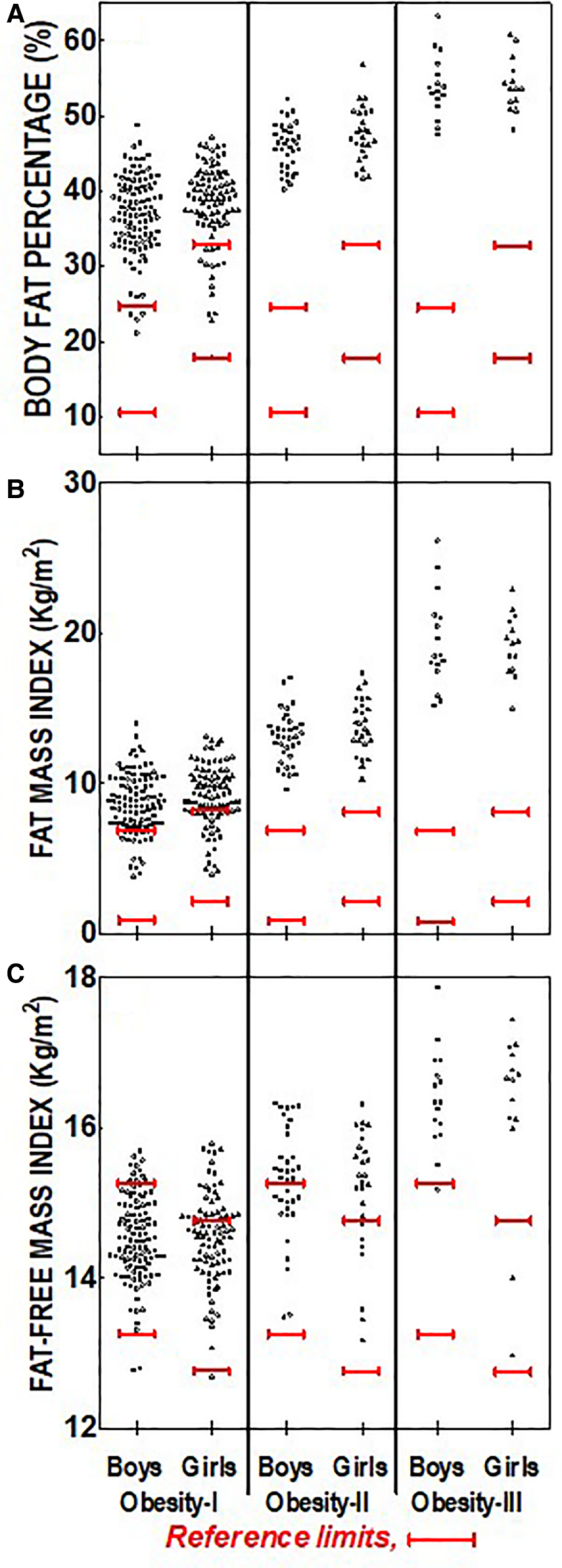
Distribution of body composition and indexes measures for the healthy weight and overweight BMI%-based weight categories for boys and girls. (**A**) Solid red lines represent the referenced (27) optimal range body fat percentage for boys (>10% to <24%) and girls (>17% to <32%); (**B**) Solid red lines represent the referenced (28) mean ± 1SD for the Fat Mass Index for boys (3.5 ± 3 kg/m2) and girls (4.8 ± 3 kg/m^2^); (**C**) solid red lines represent the referenced (28) mean ± 1SD for the Fat Free Mass Index for boys (14.2 ± 1 kg/m^2^) and girls (13.7 ± 1 kg/m^2^).

Shown in Figure 1 are comparisons of body composition data for boy and girl subjects for weight categories of HW and OW. These two categories are typically considered “low disease risk” by published guidelines (29) in the absence of other clinical assessments or symptoms. Shown in Panels 1A, 1B, and 1C are BF%, FMI, and FFMI respectively. Also presented in each of the panels of Figure 1 are normative reference lines, or cut-points of abnormal values, for each variable based on age and sex (29, 32). This allows visual comparisons of the study population relative to cut-off values and expected ranges of normal values. We have also provided information concerning our modeling of these associations in the Supplementary Tables S2A–C.

Figure 1A shows individual subject BF% for each sex and low-risk weight category respectively, with cut-points for this age, superimposed on the observed per-subject data for visualization. Of the group of boys in the HW category we observed 56 of 289 (19.4%) to have BF% above expected upper limit of normal (i.e., >24%). Boys in the OW group also were found to commonly exceed upper limit of normal BF% (58 of 90, or 64.4%, Panel A). Of the 314 girls in the HW group 12 were found to have BF% above upper limit of normal (32% for girls of this age). In contrast, a great fraction of above-BF% cutoff was observed for OW girls (46 of 85, or 54.1% of subjects).

Figure 1B shows individual subject FMI for boys vs. girls in the two low-risk weight categories. Also shown are reference values and upper and lower limits of normal (32). Nearly all of the subjects of the HW group were found to have FMI within the expected normal range. A greater incidence of above-normal range was observed in OW boys (20 of 89, 22.5%) and OW girls (16 of 85, 18.8%).

Figure 1C shows individual subject FFMI vs. reference values for boys and girls in the HW and OW groups. Notable patterns emerged in the HW category for both sexes, in that 144 of 289 boys (49.8%) and 126 of 314 (40.1%) girls in this weight category were found to have FFMI lower than the reference value cutoff suggesting a lesser fat-free mass than expected for a substantial portion of this HW group of children. A lesser incidence of lower than expected FFMI was observed in the OW group for either sex, and no significant differences in average FFMI was observed between the two weight classes, or between sexes.

**Figure 2:**
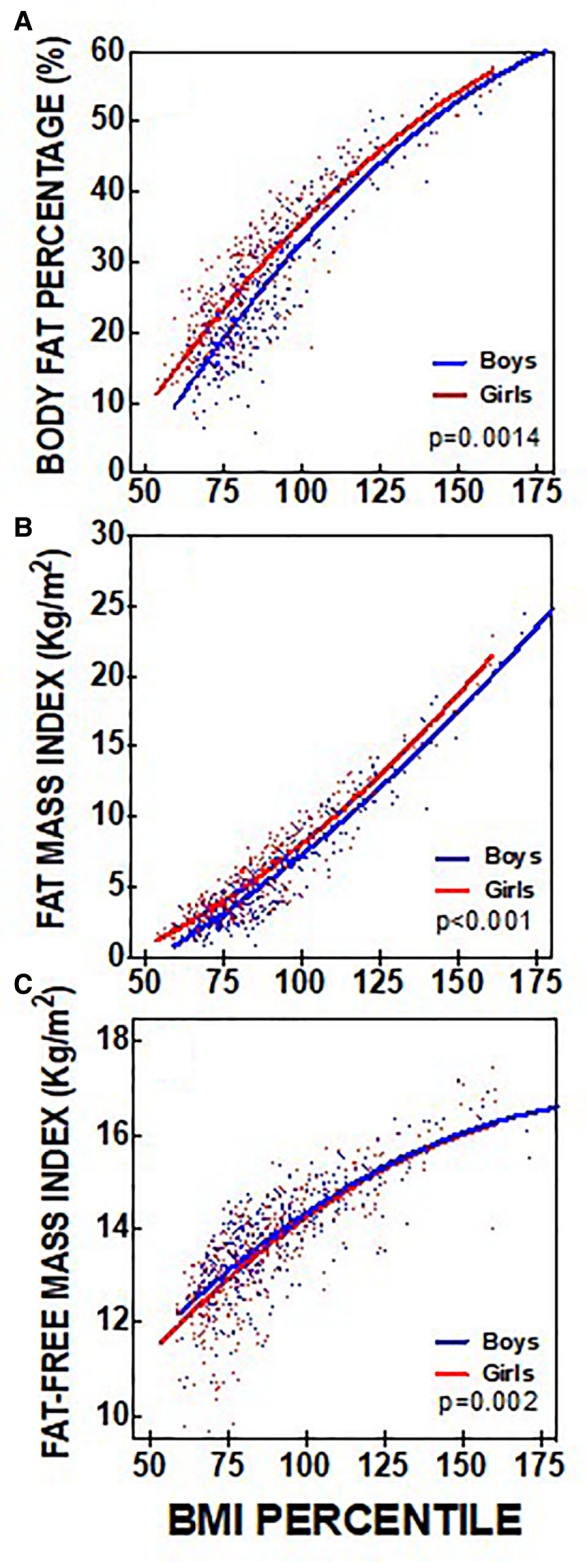
Distribution of body composition and indexes measures for the obese BMI%-based weight categories for boys and girls. (**A**) Solid red lines represent the referenced (27) optimal range body fat percentage for boys (>10% to <24%) and girls (>17% to <32%); (**B**) Solid red lines represent the referenced (28) mean ± 1SD for the Fat Mass Index for boys (3.5 ± 3 kg/m^2^) and girls (4.8 ± 3 kg/m^2^); (**C**) Solid red lines represent the referenced (28) mean ± 1SD for the Fat Free Mass Index for boys (14.2 ± 1 kg/m^2^) and girls (13.7 ± 1 kg/m^2^).

Shown in Figure 2 are body composition values (BF%, FMI, FFMI) for children categorized as Obese, and stratified to classes I, II, and III in accordance with current guidelines. Reference ranges are also superimposed on each panel. The BF% was consistently above expected values for boys and girls at each Obesity category (Figure 2A) and average values were increased at each Obesity class. In many subjects BF% was found to be more than 2-fold above upper limits. This trend was more prevalent in the OB-I and OB-II groups, wherein BF% was over 50% (this represented approximately 20 kg of fat per subject). FMI was also elevated at each Obesity class for boys and girls (Figure 2B), with no difference between sexes at each Obesity category. In the OB-I grouping 28 of a total 196 subjects (14.3%) were found to have FMI within normal range (Figure 2B).

**Figure 3:**
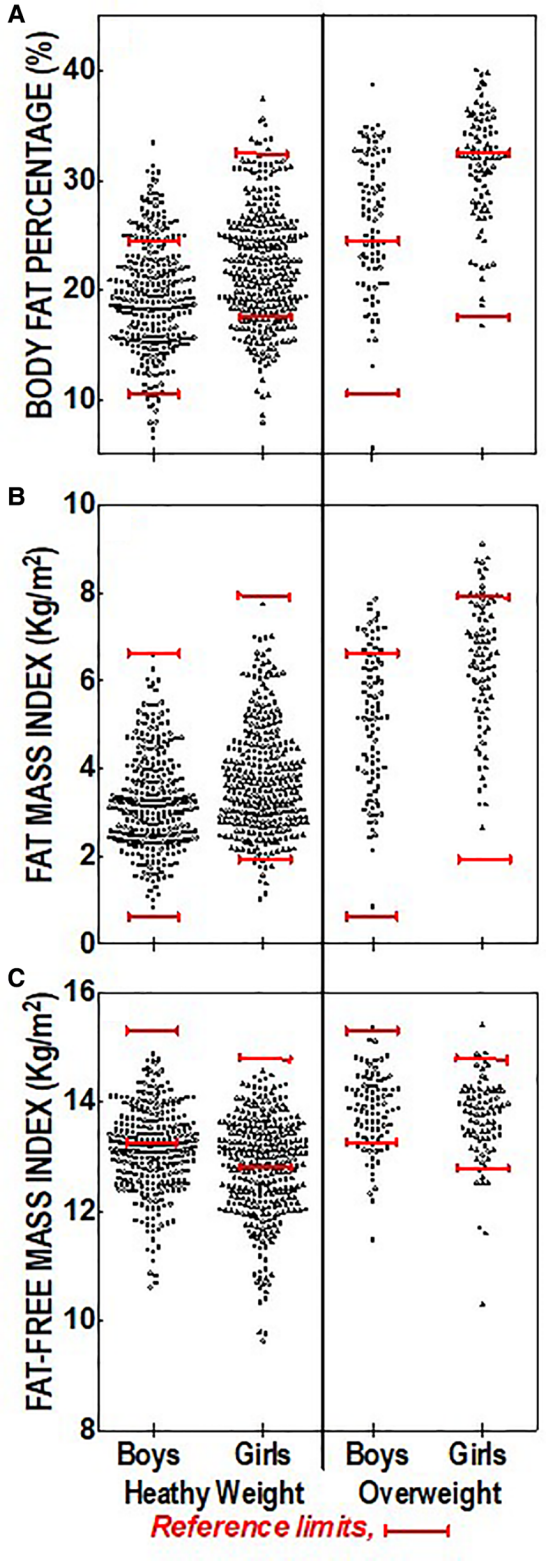
Regression analysis and relationships of BMI percentiles versus body composition and indexes with comparisons between boys and girls. (**A**) Significant correlations for boys (*r* = 0.91), girls (*r* = 0.90) and the total group (*r* = 0.89); (**B**) Significant correlations for boys (*r* = 0.93), girls (*r* = 0.92) and the total group (0.92); (**C**) Significant correlations for boys (*r* = 0.82), girls (*r* = 0.76), and the total group (*r* = 0.80). Sex slope comparison were significant for Figures 3A (*p* = 0.0014) and 3B (*p* < 0.001) and 3C (*p* = 0.002).

Figure 3 shows regression analysis and relationships of BMI% vs. determined body composition for each subject, with comparisons between boys and girls. Model fitting using a quadratic equation showed statistically significant differences between boys and girls, with a generally higher BF% and FMI at any BMI% for girls when compared to boys (Figure 3A). Regression analysis of FMI vs. BMI% also revealed a significant difference between boys and girls (Figure 3B). Similarly, regression analysis of FFMI vs. BMI% also revealed a significant difference between boys and girls (Figure 3C) however this difference was not as strong as the analyses for BF% and FMI vs. BMI%.

## Discussion

Childhood obesity has reached epidemic proportions in the US, with the fastest growth affecting younger age groups, and the increased prevalence of individuals with extreme obesity. Stratifying individual children, identifying those with highest disease risks, and development of prevention and/or treatment strategies have become of vital importance. The use of BMI in combination with large dataset normalizations can provide rough approximation of risks, but without better tools to define adiposity in early life it will be difficult to optimize strategies and evaluate treatment successes. While previous reports have indicated significant relationship between BMI% and BF% derived from BIA, using absolute and relative measures of body composition (both fat and fat-free masses) provide greater sensitivity for classification and evaluating treatment effectiveness (33, 34).

In this study we explored the potential value of BIA for capturing additional subject-specific data regarding a child's adipose status and investigated the relationships to BMI% classifications that have become commonly employed in pediatric clinical care. While more sophisticated methods may provide greater accuracy of body composition measures and indexes of children, BIA provides a safe, non-invasive, economical (cost and time; taking less than 1 min to perform) method of providing additional information in clinical and field settings if a properly validated and cross-validated BIA equation is available (30, 31). Over one thousand children were studied with no adverse event occurrences and the processes employed were well tolerated in children of 5–11 years. The large cohort of children studied were primarily from the surrounding region of Kentucky Children's Hospital and from rural counties. We found 27% of our total study cohort (30% of boys, and 24% of girls) had BMI percentiles classified as obese, well-exceeding the most recently reported (2015–2016) national average for this age group (6–11 years, 18% among all children, 20% of boys, 16% of girls) (28).

A goal of this investigation was to compare actual body composition variables to BMI% categories recommended by several agencies (CDC, AAP, others), as well as to compare subject-specific data to what are considered “appropriate” values in BF%, FMI, and FFMI based on reference publications. We considered the published values used as thresholds as reliable for identifying normal vs. abnormal body composition status (27, 32).

In examining the total cohort combined we found that as weight classification increased from HW and OW to OB I, II, and III adiposity increased (e.g., BF%, and FMI increased, see Figures 1, 2). These trends are perhaps expected and suggest that BMI% stratifications can identify the most extreme cases of adiposity. However, we found surprisingly high levels of fatness in the HW and OW groups, which are typically considered “low-risk”. For example, a large proportion of children, especially boys (19.4%), in the HW group had BF% above expected, and this was even greater in OW group (64% for boys and 54.1% girls). Whether these children are truly “low-risk” remains of question and it is possible that the BMI-based methods alone are insensitive in identifying such cases.

An additional finding in the low-risk categories was potentially important evidence of low FFMI in the HW and (to a lesser extent) OW children. It should be recognized that OB individuals often have greater fat-free mass and FFMI compared to their HW counterparts due to increased bone mineral content and muscle mass as a result of greater mechanical loading stimuli of higher body mass (35–37). Others have described “obesity related sarcopenia” and our data suggest a sarcopenic state in children that are classified as HW using standard weight classifications based on BMI%. This low FFMI may be related to the rural and young population we studied, which has been characterized as increasingly sedentary and with little access to structured physical activity (38). It is generally accepted that children with sarcopenia may experience deleterious long-term developmental and clinical outcomes (28, 39–44). Further investigation of this apparent phenomenon of sarcopenia, and its impact on disease risks during childhood, are clearly warranted.

We also observed differences in these relationships for boys vs. girls since at any BMI% level girls had a higher level of BF%, and a higher FMI (Figure 3). Others have documented sex-based differences in the incidence of obesity reporting that boys have a higher prevalence of obesity than girls (45, 46), however it should be noted that these sex differences were based on BMI% alone, and not based on BF% or FMI where the mean measures (and risk classification cutpoints) are greater for girls. Oobesity risks as well as disease outcomes and the life course and experiences of obese girls vs. boys are known to be different (47, 48). Better understanding of the mechanisms and consequences of sex-based body composition differences in early life may lead to more specific intervention and/or prevention strategies.

Mechanistic studies have shown that the primary concerning element of obesity is increased adipose tissue mass. This tissue thereby drives several physiological and biochemical adaptations that affect numerous systems. Increased vascular resistance, alterations in glucose metabolism and related hormonal systems, and enhancement of inflammatory pathways are all recognized outcomes of excess adipose tissue mass in humans of various age. Of note is that in more than half of the children, age ∼10 years, studied herein estimated fat mass was greater than 15 kg, and 20%–60% of total body weight. Furthermore, sarcopenia is recognized as a separate mechanistic driver of disease *via* reduced skeletal muscle mass and reduced insulin-sensitized glucose disposition. Thus, excess adipose tissue, decreased muscle mass, and/or their combination are important factors in obesity driven disease and better characterizing these changes in early life are needed. Prospective studies to define adipose tissue mass with respect to physiological status, disease occurrence, and/or biomarkers of disease risks are clearly warranted, as are serial measurements during child growth and development.

Some weaknesses of this study exist. For example, we focused on WNH subjects (see Methods for rationale of this focus) and the findings we present may be non-representative of other groups. In addition, we did not perform maturational assessments for our child participants and can only speculate that the majority of the participants were prepubescent. Since it has long been recognized that pubertal stage alters the densities and the proportions of the components of fat-free mass (49), the accumulation of total body fat and fat distribution (50), and may have an impact on metabolism and insulin sensitivity (51), this study limitation warrants consideration. We also focused on the specific use of BIA as the sole method of body composition assessment; our investigative team has much experience in these methods and we are a proponent of this approach since it is fast, reliable, and has potential to fit into regular pediatric clinical care workflow and can be developed as part of a specialized clinic (ongoing study). It also has an advantage of screening at schools and other sites and is convenient for use in very young children. Other methodologies and broader trials of such tools for patient care are worthy of further investigation.

## Conclusions

In summary, the childhood obesity pandemic has relied heavily on BMI-based assessments of child body status, and even with more recent adaptations using normalized percentages this approach may be inaccurately categorizing some children. The population studied showed that some children have higher fat mass, lesser muscle mass, or both, even in the categories of HW and OW; they may be less healthy than the categorical methods can identify alone and this inaccuracy may be more common in girls than boys. Incorporation of body composition assessments (especially easy and reliable and noninvasive methods like BIA) could enhance the sensitivity and specificity of risks stratifications.

## Data Availability

The raw data supporting the conclusions of this article will be made available by the authors, without undue reservation.

## References

[1] Ogden CL Carroll MD Kit BK Flegal KM. Prevalence of childhood and adult obesity in the United States, 2011–2012. JAMA. (2014) 311(8):806–14. 10.1001/jama.2014.732 24570244 PMC4770258

[2] Wang Y. Disparities in pediatric obesity in the United States. Adv Nutr. (2011) 2(1):23–31. 10.3945/an.110.000083 22211187 PMC3042789

[3] Lutfiyya MN Lipsky MS Wisdom-Behounek J Inpanbutr-Martinkus M. Is rural residency a risk factor for overweight and obesity for U.S. Children? Obesity (Silver Spring). (2007) 15(9):2348–56. 10.1038/oby.2007.278 17890504

[4] Lobstein T Jackson-Leach R Moodie ML Hall KD Gortmaker SL Swinburn BA Child and adolescent obesity: part of a bigger picture. Lancet. (2015) 385(9986):2510–20. 10.1016/S0140-6736(14)61746-3 25703114 PMC4594797

[5] Skinner AC Skelton JA. Prevalence and trends in obesity and severe obesity among children in the United States, 1999–2012. JAMA Pediatr. (2014) 168(6):561–6. 10.1001/jamapediatrics.2014.21 24710576

[6] Fryar CD CM Afful J. Prevalence of overweight, obesity, and severe obesity among children and adolescents aged 2–19 years: United States, 1963–1965 through 2017–2018. Atlanta, GA: NCHS Health E-Stats (2020). Available from: https://www.cdc.gov/nchs/data/hestat/obesity-child-17-18/obesity-child.htm

[7] Freedman DS Dietz WH Srinivasan SR Berenson GS. The relation of overweight to cardiovascular risk factors among children and adolescents: the bogalusa heart study. Pediatrics. (1999) 103(6 Pt 1):1175–82. 10.1542/peds.103.6.1175 10353925

[8] Sorof J Daniels S. Obesity hypertension in children: a problem of epidemic proportions. Hypertension. (2002) 40(4):441–7. 10.1161/01.HYP.0000032940.33466.12 12364344

[9] Barlow SE Dietz WH. Obesity evaluation and treatment: expert committee recommendations. The maternal and child health bureau, health resources and services administration and the department of health and human services. Pediatrics. (1998) 102(3):E29. 10.1542/peds.102.3.e29 9724677

[10] Daniels SR Arnett DK Eckel RH Gidding SS Hayman LL Kumanyika S Overweight in children and adolescents: pathophysiology, consequences, prevention, and treatment. Circulation. (2005) 111(15):1999–2012. 10.1161/01.CIR.0000161369.71722.10 15837955

[11] Fagot-Campagna A Pettitt DJ Engelgau MM Burrows NR Geiss LS Valdez R Type 2 diabetes among north American children and adolescents: an epidemiologic review and a public health perspective. J Pediatr. (2000) 136(5):664–72. 10.1067/mpd.2000.105141 10802501

[12] Freedman DS Mei Z Srinivasan SR Berenson GS Dietz WH. Cardiovascular risk factors and excess adiposity among overweight children and adolescents: the bogalusa heart study. J Pediatr. (2007) 150(1):12–7.e2. 10.1016/j.jpeds.2006.08.042 17188605

[13] Gidding SS Bao W Srinivasan SR Berenson GS. Effects of secular trends in obesity on coronary risk factors in children: the bogalusa heart study. J Pediatr. (1995) 127(6):868–74. 10.1016/S0022-3476(95)70020-X 8523181

[14] Geserick M Vogel M Gausche R Lipek T Spielau U Keller E Acceleration of BMI in early childhood and risk of sustained obesity. N Engl J Med. (2018) 379(14):1303–12. 10.1056/NEJMoa1803527 30281992

[15] Maring B Greenspan LC Chandra M Daniels SR Sinaiko A Prineas RJ Comparing US paediatric and adult weight classification at the transition from late teenage to young adulthood. Pediatr Obes. (2015) 10(5):371–9. 10.1111/ijpo.274 25612172 PMC5100891

[16] Vanderwall C Randall Clark R Eickhoff J Carrel AL. BMI Is a poor predictor of adiposity in young overweight and obese children. BMC Pediatr. (2017) 17(1):135. 10.1186/s12887-017-0891-z 28577356 PMC5457636

[17] Maynard LM Wisemandle W Roche AF Chumlea WC Guo SS Siervogel RM. Childhood body composition in relation to body mass index. Pediatrics. (2001) 107(2):344–50. 10.1542/peds.107.2.344 11158468

[18] Franckle R Adler R Davison K. Accelerated weight gain among children during summer versus school year and related racial/ethnic disparities: a systematic review. Prev Chronic Dis. (2014) 11:E101. 10.5888/pcd11.130355 24921899 PMC4060873

[19] Wang YCH. Use of percentiles and Z-scores in anthropometry. In: Preedy V, editors. Handbook of anthropometry springer. New York, NY: Springer. (2012). p. 29–48.

[20] Kalantari N Mohammadi NK Rafieifar S Eini-Zinab H Aminifard A Malmir H Indicator for success of obesity reduction programs in adolescents: body composition or body mass index? Evaluating a school-based health promotion project after 12 weeks of intervention. Int J Prev Med. (2017) 8:73. 10.4103/ijpvm.IJPVM_306_16 29026505 PMC5634063

[21] Dietz WH. Time to adopt new measures of severe obesity in children and adolescents. Pediatrics. (2017) 140(3). 10.1542/peds.2017-2148 28830921

[22] Gulati AK Kaplan DW Daniels SR. Clinical tracking of severely obese children: a new growth chart. Pediatrics. (2012) 130(6):1136–40. 10.1542/peds.2012-0596 23129082 PMC4528342

[23] Murphy MO Huang H Bauer JA Schadler A Makhoul M Clasey JL Impact of pediatric obesity on diurnal blood pressure assessment and cardiovascular risk markers. Front Pediatr. (2021) 9:596142. 10.3389/fped.2021.596142 33748038 PMC7969716

[24] Pietrobelli A Faith MS Allison DB Gallagher D Chiumello G Heymsfield SB. Body mass index as a measure of adiposity among children and adolescents: a validation study. J Pediatr. (1998) 132(2):204–10. 10.1016/S0022-3476(98)70433-0 9506629

[25] Dencker M Thorsson O Linden C Wollmer P Andersen LB Karlsson MK. BMI And objectively measured body fat and body fat distribution in prepubertal children. Clin Physiol Funct Imaging. (2007) 27(1):12–6. 10.1111/j.1475-097X.2007.00709.x 17204032

[26] Wright CM Sherriff A Ward SC McColl JH Reilly JJ Ness AR. Development of bioelectrical impedance-derived indices of fat and fat-free mass for assessment of nutritional status in childhood. Eur J Clin Nutr. (2008) 62(2):210–7. 10.1038/sj.ejcn.1602714 17356557

[27] Wohlfahrt-Veje C Tinggaard J Winther K Mouritsen A Hagen CP Mieritz MG Body fat throughout childhood in 2647 healthy danish children: agreement of BMI, waist circumference, skinfolds with dual x-ray absorptiometry. Eur J Clin Nutr. (2014) 68(6):664–70. 10.1038/ejcn.2013.282 24473457

[28] Hales CM Carroll MD Fryar CD Ogden CL. Prevalence of obesity among adults and youth: United States, 2015–2016. NCHS Data Brief. (2017) 288:1–8. PMID: 29155689

[29] Kuczmarski RJ Ogden CL Guo SS Grummer-Strawn LM Flegal KM Mei Z 2000 CDC growth charts for the United States: methods and development. Vital Health Stat. (2002) 11(246):1–190. PMID: 12043359

[30] Clasey JL Bradley KD Bradley JW Long DE Griffith JR. A new BIA equation estimating the body composition of young children. Obesity (Silver Spring). (2011) 19(9):1813–7. 10.1038/oby.2011.158 21681223

[31] Williams DP Going SB Lohman TG Harsha DW Srinivasan SR Webber LS Body fatness and risk for elevated blood pressure, total cholesterol, and serum lipoprotein ratios in children and adolescents. Am J Public Health. (1992) 82(3):358–63. 10.2105/AJPH.82.3.358 1536350 PMC1694353

[32] Freedman DS Wang J Maynard LM Thornton JC Mei Z Pierson RN Relation of BMI to fat and fat-free mass among children and adolescents. Int J Obes. (2005) 29(1):1–8. 10.1038/sj.ijo.0802735 15278104

[33] Mast M Langnase K Labitzke K Bruse U Preuss U Muller MJ. Use of BMI as a measure of overweight and obesity in a field study on 5–7 year old children. Eur J Nutr. (2002) 41(2):61–7. 10.1007/s003940200009 12083315

[34] Sorensen K Juul A. BMI percentile-for-age overestimates adiposity in early compared with late maturing pubertal children. Eur J Endocrinol. (2015) 173(2):227–35. 10.1530/EJE-15-0239 25979736

[35] van Leeuwen J Koes BW Paulis WD van Middelkoop M. Differences in bone mineral density between normal-weight children and children with overweight and obesity: a systematic review and meta-analysis. Obes Rev. (2017) 18(5):526–46. 10.1111/obr.12515 28273691

[36] Fintini D Cianfarani S Cofini M Andreoletti A Ubertini GM Cappa M The bones of children with obesity. Front Endocrinol. (2020) 11:200. 10.3389/fendo.2020.00200 PMC7193990 32390939

[37] Garcia-Vicencio S Coudeyre E Kluka V Cardenoux C Jegu AG Fourot AV The bigger, the stronger? Insights from muscle architecture and nervous characteristics in obese adolescent girls. Int J Obes. (2016) 40(2):245–51. 10.1038/ijo.2015.158 26285605

[38] Smith LH Petosa RL Laurent D. Efficacy of “mentoring to be active” on weight loss, body mass Index, and body fat among obese and extremely obese youth in rural appalachia. J Rural Health. (2020) 36(1):77–87. 10.1111/jrh.12410 31885129 PMC7185163

[39] Ooi PH Thompson-Hodgetts S Pritchard-Wiart L Gilmour SM Mager DR. Pediatric sarcopenia: a paradigm in the overall definition of malnutrition in children? JPEN J Parenter Enteral Nutr. (2020) 44(3):407–18. 10.1002/jpen.1681 31328301

[40] Suzuki D Kobayashi R Sano H Hori D Kobayashi K. Sarcopenia after induction therapy in childhood acute lymphoblastic leukemia: its clinical significance. Int J Hematol. (2018) 107(4):486–9. 10.1007/s12185-017-2388-9 29234981

[41] Mager DR Carroll MW Wine E Siminoski K MacDonald K Kluthe CL Vitamin D status and risk for sarcopenia in youth with inflammatory bowel diseases. Eur J Clin Nutr. (2018) 72(4):623–6. 10.1038/s41430-018-0105-2 29391593

[42] Kim K Hong S Kim EY. Reference values of skeletal muscle mass for Korean children and adolescents using data from the Korean national health and nutrition examination survey 2009–2011. PLoS One. (2016) 11(4):e0153383. 10.1371/journal.pone.0153383 27073844 PMC4830599

[43] Lurz E Patel H Frimpong RG Ricciuto A Kehar M Wales PW Sarcopenia in children with end-stage liver disease. J Pediatr Gastroenterol Nutr. (2018) 66(2):222–6. 10.1097/MPG.0000000000001792 29356766

[44] Steffl M Chrudimsky J Tufano JJ. Using relative handgrip strength to identify children at risk of sarcopenic obesity. PLoS One. (2017) 12(5):e0177006. 10.1371/journal.pone.0177006 28542196 PMC5441624

[45] Shah B Tombeau Cost K Fuller A Birken CS Anderson LN. Sex and gender differences in childhood obesity: contributing to the research agenda. BMJ Nutr Prev Health. (2020) 3(2):387–90. 10.1136/bmjnph-2020-000074 33521549 PMC7841817

[46] Yusuf ZI Dongarwar D Yusuf RA Bell M Harris T Salihu HM. Social determinants of overweight and obesity among children in the United States. Int J MCH AIDS. (2020) 9(1):22–33. 10.21106/ijma.337 32123625 PMC7031877

[47] Lee JM Pilli S Gebremariam A Keirns CC Davis MM Vijan S Getting heavier, younger: trajectories of obesity over the life course. Int J Obes. (2010) 34(4):614–23. 10.1038/ijo.2009.235 PMC2926791 19949415

[48] Huebschmann AG Huxley RR Kohrt WM Zeitler P Regensteiner JG Reusch JEB. Sex differences in the burden of type 2 diabetes and cardiovascular risk across the life course. Diabetologia. (2019) 62(10):1761–72. 10.1007/s00125-019-4939-5 31451872 PMC7008947

[49] Roemmich JN Clark PA Weltman A Rogol AD. Alterations in growth and body composition during puberty. I. Comparing multicompartment body composition models. J Appl Physiol (1985). (1997) 83(3):927–35. 10.1152/jappl.1997.83.3.927 9292482

[50] Loomba-Albrecht LA Styne DM. Effect of puberty on body composition. Curr Opin Endocrinol Diabetes Obes. (2009) 16(1):10–5. 10.1097/MED.0b013e328320d54c 19115520

[51] Travers SH Jeffers BW Bloch CA Hill JO Eckel RH. Gender and tanner stage differences in body composition and insulin sensitivity in early pubertal children. J Clin Endocrinol Metab. (1995) 80(1):172–8. 10.1210/jcem.80.1.7829608 7829608

