# Supplementary material for: Body mass index percentiles versus body composition assessments: Challenges for disease risk classifications in children
Source: Front Pediatr. 2023 Mar 3;11:1112920. doi: 10.3389/fped.2023.1112920 (PMC10020489; doi:10.3389/fped.2023.1112920)
Supplement: Supplementary file 1 [file Table1.docx]

**Table 1. Age, Anthropometric and Body Composition Measures and Indexes by Sex and BMI Percentile Category.**

|  | **Boys** | | | | | |
| --- | --- | --- | --- | --- | --- | --- |
|  | **Healthy (n=288)**  **Mean ± SE**  **(range)** | **Overweight (n=90)**  **Mean ± SE**  **(range)** | **Obese I (n=108)**  **Mean ± SE**  **(range)** | **Obese II (n= 35)**  **Mean ± SE**  **(range)** | **Obese III (n=16)**  **Mean ± SE**  **(range)** | **Total (n=537)**  **Mean ± SE**  **(range)** |
| **Age**  **(yrs)** | 9.6 ± 0.1  (5.0-11.7) | 8.8 ± 0.2**^a^**  (5.0-11.9) | 9.8 ± 0.1  (5.9-11.9) | 10.0 ± 0.2  (5.6-11.9) | 10.5 ± 0.2**^a^**  (9.0-11.9) | 9.6 ± 0.1  (5.0-11.9) |
| **Body Weight**  **(kg)** | 31.5 ± 0.4  (16.5-53.9) | 36.0 ± 1.0**^a^**  (17.8-65.1) | 49.3 ± 1.1**^a,b^**  (21.4-72.6) | 62.6 ± 2.0**^a,b,c^**  (34.3-84.2) | 82.9 ± 2.8**^a,b,c,d^**  (66.1-101.8) | 39.4 ± 0.6  (16.5-101.8) |
| **Height**  **(cm)** | 137.8 ± 0.6  (109.6-165.9) | 136.2 ± 1.4  (103.8-172.3) | 144.7 ± 1.1**^a,b^**  (110.3-162.1) | 147.8 ± 1.7**^a,b^**  (116.2-168.6) | 151.9 ± 2.5**^a,b,c^**  (139.5-169.8) | 140.0 ± 0.5  (103.8-172.3) |
| **BMI**  **(kg/m^2^)** | 16.4 ± 0.1  (13.1-20.4) | 18.9 ± 0.2**^a^**  (15.3-22.3) | 23.2 ± 0.2^a,b^  (17.6-28.8) | 28.4 ± 0.4**^a,b,c^**  (23.9-33.1) | 39.8 ± 0.8**^a,b,c,d^**  (31.5-41.4) | 19.5 ± 0.2  (13.1-41.4) |
| **BMI Percentile**  **(%)** | 75.4 ± 0.4  (59.2-84.9) | 90.2 ± 0.3**^a^**  (85.2-94.9) | 105.6 ± 0.7**^a,b^**  (95.1-119.9) | 128.4 ± 0.9**^a,b,c^**  (120.5-139.9) | 159.0 ± 3.6**^a,b,c,d^**  (141.3-185.5) | 89.9 ± 0.9  (59.2-185.5) |
| **Fat Mass**  **(kg)** | 6.3 ± 0.2  (1.3-18.0) | 10.0 ± 0.5**^a^**  (1.2-21.3) | 18.6 ± 0.6**^a,b^**  (4.9-35.1) | 29.0 ± 1.2**^a,b,c^**  (14.3-42.7) | 44.9 ± 2.0**^a,b,c,d^**  (31.4-60.5) | 12.0 ± 0.4  (1.2-60.5) |
| **Fat-Free Mass**  **(kg)** | 25.2 ± 0.3  (14.6-36.6) | 26.0 ± 0.6  (14.1-43.8) | 30.7 ± 0.4**^a,b^**  (16.5-39.8) | 33.6 ± 0.9**^a,b,c^**  (20.1-44.0) | 38.0 ± 1.3**^a,b,c,d^**  (29.5-47.8) | 27.4 ± 0.3  (14.1-47.8) |
| **Percentage Fat**  **(%)** | 19.3 ± 0.3  (6.4-33.5) | 26.3 ± 0.6**^a^**  (5.6-38.8) | 40.5 ± 0.5**^a,b^**  (21.2-48.8) | 45.9 ± 0.5**^a,b,c^**  (40.3-52.2) | 54.0 ± 1.0**^a,b,c,d^**  (47.5-63.3) | 26.8 ± 0.5  (5.6-63.3) |
| **FM Index**  **(kg/m^2^)** | 3.2 ± 0.1  (10.6-14.9) | 5.7 ± 0.2**^a^**  (0.8-26.2) | 8.6 ± 0.2**^a,b^**  (3.8-14.0) | 13.1 ± 0.3**^a,b,c^**  (9.6-17.1) | 19.5 ± 0.8**^a,b,c,d^**  (15.2-26.2) | 5.7 ± 0.2  (0.8-26.2) |
| **FFM Index**  **(kg/m^2^)** | 13.1 ± 0.0  10.6-17.9) | 26.0 ± 0.6**^a^**  (10.6-17.9) | 14.5 ± 0.1**^a,b^**  (12.8-15.7) | 15.3 ± 0.1**^a,b,c^**  (13.5-16.3) | 16.4 ± 0.2**^a,b,c,d^**  (15.2-17.9) | 13.8 ± 0.0  (10.6-17.9) |
|  | **Girls** | | | | | |
|  | **Healthy (n=314)**  **Mean ± SE**  **(range)** | **Overweight (n=85)**  **Mean ± SE**  **(range)** | **Obese I (n=88)**  **Mean ± SE**  **(range)** | **Obese II (n= 26)**  **Mean ± SE**  **(range)** | **Obese III (n=14)**  **Mean ± SE**  **(range)** | **Total (n=528)**  **Mean ± SE**  **(range)** |
| **Age**  **(yrs)** | 9.7 ± 0.1  (5.2-11.9) | 9.4 ± 0.2*  (5.0-11.8) | 9.8 ± 0.2  (5.0-11.9) | 9.8 ± 0.1  (7.8-10.9) | 10.2 ± 0.2  (9.1-11.6) | 9.7 ± 0.1  (5.0-11.9) |
| **Body Weight**  **(kg)** | 32.1 ± 0.4  (14.4-55.6) | 39.8 ± 1.0***^,a^**  (15.5-58.7) | 50.0 ± 1.1**^a,b^**  (22.8-78.2) | 64.1 ± 1.9**^a,b,c^**  (50.3-82.0) | 78.8 ± 3.5**^a,b,c,d^**  (59.5-105.5) | 39.1 ± 0.6  (14.4-105.5) |
| **Height**  **(cm)** | 137.9 ± 0.7  (105.8-166.5) | 139.4 ± 1.4  (99.4-164.5) | 143.8 ± 1.2**^a,b^**  (114.0-168.5) | 148.0 ± 1.7**^a,b^**  (128.2-161.7) | 148.6 ± 3.0**^a,b^**  (124.4-166.0) | 139.9 ± 0.5  (99.4-168.5) |
| **BMI**  **(kg/m^2^)** | 16.6 ± 0.1  (12.8-20.8) | 19.5 ± 0.2***^,a^**  (12.8-39.7) | 23.8 ± 0.2**^a,b^**  (17.5-28.6) | 29.1 ± 0.4**^a,b,c^**  (24.6-32.4) | 35.5 ± 0.6**^a,b,c,d^**  (31.2-39.7) | 19.5 ± 0.2  (12.8-39.7) |
| **BMI Percentile**  **(%)** | 73.4 ± 0.4*  (53.7-84.9) | 90.0 ± 0.3**^a^**  (85.1-94.9) | 104.9 ± 0.8**^a,b^**  (95.1-119.9) | 128.7 ± 1.3**^a,b,c^**  (120.7-139.7) | 153.3 ± 1.5**^a,b,c,d^**  (143.1-160.6) | 86.2 ± 0.9*  (53.7-160.6) |
| **Fat Mass**  **(kg)** | 7.4 ± 0.2*  (1.7-17.7) | 12.8 ± 0.5***^,a^**  (3.8-22.6) | 19.6 ± 0.7**^a,b^**  (5.2-36.1) | 30.6 ± 1.1**^a,b,c^**  (21.4-41.8) | 42.7 ± 2.0^a,b,c,d^  (32.5-57.4) | 12.4 ± 0.4  (1.7-57.4) |
| **Fat-Free Mass**  **(kg)** | 24.7 ± 0.3  (10.8-37.9) | 27.0 ± 0.6**^a^**  (10.2-37.4) | 30.3 ± 0.5**^a,b^**  (17.3-42.1) | 33.4 ± 1.0**^a,b,c^**  (21.7-42.0) | 36.1 ± 1.8**^a,b,c^**  (23.7-48.1) | 26.8 ± 0.3  (10.2-48.1) |
| **Percentage Fat**  **(%)** | 22.3 ± 0.3*  (8.0 – 37.4) | 31.3 ± 0.6***^,a^**  (16.8-40.2) | 38.4 ± 0.6**^a,b^**  (22.8-47.3) | 47.7 ± 0.7***^,a,b,c^**  (41.8-56.9) | 54.2 ± 1.0a,b,c,d  (48.3-60.9) | 28.5 ± 0.4*  (8.0-60.9) |
| **FM Index**  **(kg/m^2^)** | 3.8 ± 0.1*  (1.0-7.8) | 6.4 ± 0.2***^,a^**  (2.6-9.1) | 9.2 ± 0.2**^a,b^**  (4.0-13.2) | 13.9 ± 0.4**^a,b,c^**  (10.3-17.4) | 19.3 ± 0.5**^a,b,c,d^**  (15.1-23.0) | 6.0 ± 0.2  (1.0-23.0) |
| **FFM Index**  **(kg/m^2^)** | 12.8 ± 0.1*  (9.7-14.6) | 13.7 ± 0.1**^a^**  (10.3-15.4) | 14.6 ± 0.1**^a,b^**  (12.7-15.8) | 15.2 ± 0.2**^a,b,c^**  (13.2-16.3) | 16.2 ± 0.3**^a,b,c,d^**  (13.0-17.4) | 13.5 ± 0.1*  (9.7-17.5) |

**Obese I = Obese Class I; Obese II = Obese Class II; Obese III = Obese Class III; BMI = Body Mass Index; BMI Percentile = BMI for Age and Sex Percentile; FM Index = Fat Mass Index; FFM Index = Fat Free Mass Index**

****p* < 0.05 versus boys of same BMI for age and sex category**

^a^**p < 0.05 versus Healthy category for the same sex**

**^b^p < 0.05 versus Overweight for the same sex category**

**^c^p < 0.05 versus Obese Class I for the same sex category**

**^d^p < 0.05 versus Obese Class II for the same sex category**
