# Supplementary material for: Body mass index percentiles versus body composition assessments: Challenges for disease risk classifications in children
Source: Front Pediatr. 2023 Mar 3;11:1112920. doi: 10.3389/fped.2023.1112920 (PMC10020489; doi:10.3389/fped.2023.1112920)
Supplement: Supplementary file 2 [file Table2.docx]

**Supplemental Tables:**

**Table 2A. Model for Body Fat Percentage**.

| **Parameter** | **Estimate** | **p-value** |
| --- | --- | --- |
| Intercept | -32.149 | < 0.001 |
| Gender (Female) | 3.615 | < 0.001 |
| BMI Percentile | 0.817 | < 0.001 |
| (BMI Percentile)^2^ | -0.002 | < 0.001 |

**Table 2B. Model for Fat Mass Index.**

| **Parameter** | **Estimate** | **p-value** |
| --- | --- | --- |
| Intercept | -5.073 | < 0.001 |
| Gender (Female) | 0.892 | < 0.001 |
| BMI Percentile | 0.067 | < 0.001 |
| (BMI Percentile)^2^ | 0.0006 | < 0.001 |

**Table 2C. Model for Fat Free Mass Index.**

| **Parameter** | **Estimate** | **p-value** |
| --- | --- | --- |
| Intercept | 7.555 | < 0.001 |
| Gender (Female) | -0.136 | 0.002 |
| BMI Percentile | 0.090 | < 0.001 |
| (BMI Percentile)^2^ | -0.0002 | < 0.001 |
